# An efficient assignment of multiple agricultural machinery tasks based on Chaotic Cauchy Elite Variable Snake Optimization Algorithm

**DOI:** 10.1371/journal.pone.0337219

**Published:** 2025-12-12

**Authors:** Ruoxue Xiang, Xiang Liu, Min Tian, Chenglong Ban, Rongxuan Li, Jie Zhou

**Affiliations:** 1 College of Mechanical and Electrical Engineering, Shihezi University, Shihezi, China; 2 College of Information Science and Technology, Shihezi University, Shihezi, China; SR University, INDIA

## Abstract

Task allocation for agricultural machinery constitutes a critical challenge in multi-machine coordination within unmanned smart farms. Enhancing the efficiency of task allocation remains an urgent research problem. Current machinery often suffers from inefficient allocation strategies and unprocessed field areas, which lead to reduced productivity and unnecessary resource consumption. This study develops a novel task allocation model incorporating machine speed, turning time, and fuel consumption to overcome these limitations. In addition, a Chaotic Cauchy Elite Variation Snake Optimisation Algorithm (CCEVSOA) is introduced. Specifically, the algorithm employs a chaotic operator tailored for multi-machine coordination scenarios, ensuring a more uniform distribution of initial solutions across the search space. Moreover, integrating an enhanced Cauchy operator with an elite evolution strategy enlarges the search domain and mitigates premature convergence, reducing overall operation time and improving coordination efficiency. Extensive experiments verify that CCEVSOA achieves superior performance with a markedly faster convergence rate. When compared with the Snake Optimization Algorithm (SO), Genetic Algorithm (GA), Clone Selection Algorithm (CSA), Whale Optimization Algorithm (WOA), and the Improved Buzzard Evolution Algorithm based on Lévy Flight and Simulated Annealing (IBES), CCEVSOA reduces collaborative task allocation time by 103, 89, 106, 97, and 36 minutes, corresponding to efficiency improvements of 14.25%, 12.55%, 14.6%, 13.53%, and 5.5%, respectively. These findings demonstrate that optimising multi-machine task allocation through CCEVSOA yields more rational and economically efficient distribution schemes for agricultural machinery, effectively enhancing productivity while minimising resource wastage.

## Introduction

Smart agriculture embodies an advanced paradigm of modern farming, propelled by the rapid advancements in next-generation information technology and artificial intelligence. The seamless integration of these technologies into agricultural practices catalyzes the evolution toward digital and intelligent farming [1]. Within this transformative landscape, unmanned farms are becoming a cornerstone of smart agriculture, serving as a critical element in its full [2].

Farms that are unmanned are crucial to the development of smart agriculture. Through remote control of farm equipment, machinery, and facilities, or through autonomous decisions and tasks performed by intelligent devices and robots, agricultural production and operational efficiency can be enhanced [3]. The multi-machine collaboration technology employed within unmanned farms is crucial for optimizing farm machinery utilization and production efficiency at the cluster scale [4]. A persistent challenge in this context is how to effectively allocate tasks to the most suitable farm machines before fleet operation to maximize overall fleet efficiency and productivity—a problem that has long impeded the progress of smart [5].

Research on collaborative multi-robot task allocation has primarily concentrated on fields such as unmanned aerial vehicles (UAVs) and general robotics, while comparatively limited attention has been directed toward agricultural machinery [6]. Existing studies predominantly explore multi-robot task allocation within agricultural contexts, where tasks are distributed among multiple robots based on predefined evaluation criteria, with the goal of maximizing overall efficiency while ensuring the successful completion of all tasks. This approach is equally relevant to the allocation of tasks among agricultural machinery [7].

Task allocation algorithms have made some progress. The primary methods encompass approaches based on mathematical programming, heuristic search, swarm intelligence, and market mechanisms [8]. Among these, heuristic algorithms and swarm intelligence algorithms have gained widespread adoption by researchers for optimal task allocation owing to their efficiency, flexibility, and broad applicability [9]. Prominent examples include the Genetic Algorithm (GA) [10], Clonal Selection Algorithm (CSA), Whale Optimization Algorithm (WOA) [11], Particle Swarm Optimization (PSO) [12], and Snake Optimization Algorithm (SO) [13].

Although the optimization algorithms can yield effective solutions for specific problems, they frequently encounter challenges when applied to multi-machine cooperative task allocation within the same plot. It is common for agricultural machinery to be inefficient and waste resources due to problems such as slow convergence speeds, a tendency to become trapped in local optima, and suboptimal task allocation.

In order to overcome these challenges, this paper proposes an adaptive Chaotic Corsi Elite Variable Snake Optimization Algorithm (CCEVSOA) that can be used to allocate tasks among multiple machines on the same piece of farmland together. Applying this algorithm effectively reduces operation time in multi-machine cooperative tasks and significantly improves operational efficiency, thereby enhancing overall economic benefits.

The main contributions of this paper can be summed up as below:

(1) This paper describes a novel collaborative multi farm machine task allocation method which utilizes CCEVSOA. The proposed algorithm effectively reduces collaborative operation time and substantially enhances operational efficiency, leading to lower operational costs and improved economic benefits. By integrating various strategies, CCEVSOA exhibits significant advantages in optimization capability and adaptability within multi-machine collaboration scenarios, providing an efficient solution for task allocation in the same field.

(2) A novel Cauchy variation operator is introduced, broadening the solution search range and significantly reducing the likelihood of converging to local optima. Additionally, an elite evolutionary strategy is proposed to enhance the global search capability of CCEVSOA, markedly improving the algorithm’s efficiency and convergence speed. Incorporating a chaotic mapping function ensures a more uniform distribution of initial solutions within the search space, thereby enhancing solution quality and mitigating local optima convergence risk. These innovative operational and optimization strategies significantly upgrade the CCEVSOA’s capabilities of the solution, enabling it to effectively address the challenges of task allocation in multi-machine collaboration scenarios.

(3) A novel task allocation model for agricultural machinery is developed, which is comprehensive and highly accurate, effectively reflecting the actual task distribution in unmanned farms. By incorporating different machines’ heterogeneous speeds, turning times, and fuel consumption rates, a new fitness function is designed to jointly optimise operation time and fuel consumption, satisfying the requirements of multi-machine coordination in unmanned farming scenarios.

A series of simulation experiments was conducted to evaluate the performance of CCEVSOA in multi-vehicle cooperative operation scenarios. Under varying conditions—such as the number of agricultural machines, operating speeds, and field row spacing—the proposed CCEVSOA was benchmarked against SO, GA, CSA, WOA, and IBES in terms of cooperative operation time, fuel consumption, load fairness, and coordination efficiency. For example, when the number of machines was set to five and the number of field ridges was 300, the collaborative operation time achieved by CCEVSOA was 620 min. Compared with SO, GA, CSA, WOA, and IBES, this corresponded to time savings of 103, 89, 106, 97, and 36 minutes, respectively, which translate into efficiency improvements of 14.25%, 12.55%, 14.6%, 13.53%, and 5.5%. These findings demonstrate that optimising multi-machine task allocation with CCEVSOA—by jointly accounting for operation time and fuel consumption—yields the lowest fitness values, thereby reducing operational costs and enhancing overall economic benefits.

The remainder of this paper is structured as follows:

Related work reviews relevant studies on task allocation. Materials and methods introduces the task allocation model for agricultural machinery. Theory and Calculations details the design of the CCEVSOA, addressing the task allocation problem in agricultural machinery. Results presents the simulation experiments and discusses their results. Finally, Conclusion provides a summary of the paper.

## Related work

With the rapid advancement of smart agriculture, the issue of multi-copter cooperative task allocation in unmanned farms has garnered increasing attention [14]. The agricultural environment presents a complex and dynamic scenario where multi-machine cooperative task allocation constraints vary across different situations. Consequently, distinct task allocation schemes are required to address these varying constraints effectively [15].

Multi-machine cooperative task allocation involves establishing evaluation indices for agricultural machines, assigning tasks to specific machines, and maximizing the group’s overall profitability while ensuring all tasks are completed [16].

In agriculture, this task allocation can be categorized into multi-machine cooperative task allocation within the same field and multi-field task allocation. Although there has been substantial research in agricultural machine task allocation, with various algorithms and models proposed to address optimization problems, most studies have focused on task allocation techniques for robots and drones rather than agricultural machines [17]. Task allocation methods primarily include those based on mathematical planning, market mechanisms, group intelligence, and heuristic search. Among these, group intelligence algorithms and heuristic algorithms are most commonly employed by researchers due to their robust global search capabilities and adaptability to complex environments.

Mathematical planning involves solving optimization problems using mathematical theory, and branches and bounds and dynamic programming are two of the most commonly used methods. Gu et al. [18] proposed a method based on dynamic programming and approximate dynamic programming for solving the dynamic slot allocation problem in spot container transportation considering random arrivals and cancellations in order to maximize the expected profit of a shipping company. However, the algorithm faces limitations when applied to larger-scale problems due to the increased dimensionality of the variables, which results in higher computational complexity.

Market mechanism-based task allocation employs methods analogous to human auction systems to facilitate task distribution. Common approaches include contract network algorithms and auction algorithms. For instance, Zhen et al [19]. based on the contract net protocol, an improved collaborative target allocation method was proposed to address the challenge of heterogeneity and uneven allocation within UAV clusters during target attack missions. However, the algorithm suffers from slow convergence and high computational complexity. A hybrid improved contract net algorithm was developed by Zhao et al. [20] that incorporates mental coefficients, a blackboard model, and a buffer pooling mechanism to address the collaborative task allocation problem in heterogeneous multi-unmanned platforms. However, despite these improvements, the algorithm is prone to converging to local optima, which limits its overall performance.

Heuristic algorithms are designed to provide feasible solutions to problems at an acceptable cost, with commonly used methods including forbidden search algorithms, simulated annealing, and genetic algorithms. To solve the cooperative assignment problem in a maritime combat environment, Wang et al. [21] proposed a two-stage greedy auction algorithm. However, this algorithm is prone to premature convergence, which limits its effectiveness in dynamic settings. In a similar vein, Ye et al [22] introduced a two-stage task allocation method which uses an improve genetic algorithm to solve the coordinated task allocation problem in multi-robot constructional tasks. While this approach demonstrates promise, it suffers from high computational complexity when applied to large-scale tasks. Furthermore, Xiang et al. [23]developed an improved hybrid algorithm combining an enhanced genetic algorithm with simulated annealing for tackling multi-objective scheduling problems of multiple automated guided vehicles. Nevertheless, under different conditions, this method tends to fall into a local optimum and its performance is strongly influenced by the cooling rate.

In recent years, numerous enhanced meta-heuristic approaches have been developed based on traditional heuristic algorithms to improve their performance and robustness in addressing complex optimisation problems. For instance, Myriam et al. [24] combined particle swarm optimisation with the Al-Biruni Earth radius optimisation method to propose a hybrid meta-heuristic algorithm successfully applied to oral cancer detection. Although the method significantly improved classification accuracy, its convergence speed remained limited in high-dimensional feature spaces. Similarly, El-Kenawy et al. [25] employed meta-heuristic optimisation for weed detection in wheat images captured by drones, thereby confirming the potential of such methods in agricultural image recognition. Nevertheless, their adaptability to dynamic environments still requires further enhancement. Atteia et al. [26] introduced an adaptive dynamic Dipper Throated optimisation algorithm for feature selection in medical data, effectively reducing computational overhead while maintaining classification accuracy. To address the tendency of single algorithms to converge to local optima, Abdelhamid et al. [27] integrated the sine–cosine algorithm with Dipper Throated optimisation, thereby establishing a hybrid feature selection framework with enhanced global search capability. Furthermore, Alkanhel et al. [28] incorporated hybrid heuristic optimisation methods into network intrusion detection, significantly improving detection accuracy and generalisation capability through feature selection. However, computational efficiency remained suboptimal when handling large-scale datasets. Although the above studies have demonstrated strong optimisation capabilities in medical diagnosis, agricultural image recognition, and cybersecurity, their applications have predominantly focused on feature selection and pattern recognition. Research specifically targeting multi-machine collaborative task allocation in agriculture remains scarce.

Population intelligence algorithms aim to find near-optimal solutions for task allocation within a short time frame through message propagation and population optimization. A particle swarm optimization method and an ant colony optimization method are common methods. Yan et al [29] proposed a hyper-heuristic algorithm combining the influence diffusion model with particle swarm optimization for solving the problem of functional task selection and assignment in multi-robot task allocation. However, the algorithm exhibits high complexity, particularly when applied to large-scale problems, which may limit its scalability and practical applicability. Cao et al. [30] proposed an improved ant colony algorithm that reduces the operation cycle for multiple farm machines and farmlands but exhibits higher complexity than other algorithms. Wang et al [31] designed a UAV swarm task allocation algorithm based on the bionic wolf pack method to solve the dynamic task allocation problem in complex scenarios. Yun et al. examined task allocation algorithms for multi-checking time systems by considering task deadlines and energy consumption and employing prioritization to select reasonable scheduling strategies. However, local optima can occur. Guo et al [32] present a collaborative discrete bee colony algorithm for solving the problem of task allocation and scheduling of multiple agricultural robots in a smart farm. However, this algorithm exhibits high complexity, which may hinder its scalability and practical implementation in large-scale systems. The characteristics and limitations of the studies mentioned above are summarized and analyzed, leading to the following overall conclusions:

(1) Most task allocation algorithms have been predominantly utilized in fields such as UAVs and robotics, with comparatively limited application to agricultural machinery.

(2) A common limitation of these algorithms is their tendency to converge on local optima. During the search for optimal solutions, inherent constraints or initial conditions may impede the identification of global optima. This limitation can result in suboptimal task assignments in multi-machine collaborations, increasing costs and resource wastage.

(3) Algorithms frequently exhibit slow convergence and high computational complexity. Addressing multi-task allocation often involves extensive computational steps and complex mathematical operations, which escalate computational costs and reduce the efficiency of multi-task allocation in agricultural machinery. This adversely impacts the overall system’s response speed, real-time performance, and the benefits to agricultural production. Therefore, designing an algorithm specifically tailored for agricultural machinery that effectively avoids local optima while ensuring low computational complexity and rapid convergence is essential. We present a novel approach to task allocation for multiple agricultural machines, based on the CCEVSOA. This method provides a more rational task allocation scheme, significantly reducing cooperative operation time among machines. The CCEVSOA enhances the quality of initial solutions by integrating a chaos operator, expands the solution search space with a Cauchy variation operator to prevent local optima convergence, and employs an elite evolution strategy to bolster the algorithm’s global search capability.

CCEVSOA demonstrates faster convergence speed and greater robustness than other task allocation methods. It effectively generates reasonable task allocation schemes for varying numbers of agricultural machines, thereby avoiding idle and overloaded machines. This approach minimizes wasted working time, enhances operational efficiency, reduces costs, and promotes optimal resource utilization, contributing to the advancement of the agricultural economy.

## Materials and methods

### Task allocation strategy

Multi-agricultural machine cooperative operations aim to achieve optimal task allocation while adhering to operational constraints. This ensures that each agricultural machine efficiently serves designated farm plots, thereby reducing overall system execution costs, enhancing operational efficiency, and facilitating the scheduling and management of cooperative operations within the same farmland region. The primary objective is to minimize execution costs.

The task of cooperative operations within the same field involves assigning different field plots to each agricultural machine. Swarm intelligence algorithms are employed to adjust task allocations, aiming to effectively minimize the total operation time.

This paper focuses on the rational allocation of different numbers of rows in farmland to multiple agricultural machines, aiming to minimize both the time required for each machine to complete its designated tasks and the associated fuel consumption. In doing so, it seeks to optimize the efficiency of multi-machine cooperative operations and reduce resource wastage.

### Task allocation model

A mapping relationship between multiple machines and ridges of farmland is crucial to effectively allocating tasks to agricultural machinery. This ensures tasks are allocated optimally, minimizing scheduling costs and operational cycles. Such optimization enables agricultural machinery to serve farmland systematically and facilitates cooperative operations among multiple machines within the same area.

This paper addresses the rational allocation of tasks for multiple agricultural machines within a single field by developing a task allocation model. This model evaluates the operational processes of agricultural machinery and abstracts the complexities involved in multi-machine cooperative operations.

An essential step in task allocation is constructing an environment map that describes the working environment using known environmental data. This map translates real-world environmental features into recognizable and storable map information that agricultural machinery can use effectively.

To facilitate model construction, this paper assumes a fixed number of ridges in the farmland area, requiring agricultural machinery to complete cultivation on all ridges—achieving full-coverage path planning under optimal task allocation. The heterogeneity of agricultural machinery is considered in two aspects: first, variations in straight-line working speed and turning time among different machines; second, differing fuel consumption rates during operations. Due to this heterogeneity, total operation time is no longer solely determined by straight-line travel distance; turning time and fuel constraints also become critical factors. Different task allocation schemes result in varying numbers of turns and operational distances for each machine, directly impacting total operation time and fuel consumption.

Under the aforementioned conditions, the task allocation problem for multi-machine cooperative operations can be formulated as a multi-objective optimization problem. Specifically, it involves determining a rational distribution of crop rows among different agricultural machines. The primary objectives are to minimize the total completion time for all machines and to reduce overall fuel consumption, thereby lowering operational costs. An integrated task allocation and path planning model for multi-machine cooperative operations is proposed to address this challenge, grounded in actual field conditions. The model comprehensively accounts for key factors, including the number of field ridges, machines, each machine’s straight-line working speed, turning time characteristics, and fuel consumption rates during straight-line and turning operations. The task allocation model operates according to the following rules.

Meanwhile, the rules governing the task allocation model are as follows:

(1) The agricultural machinery starts its operation from beneath the field mounds, traveling in a direction from bottom to top. Each agricultural machine operates at a different task execution speed, independently and without influence from others.

(2) Agricultural machinery operations adopt the A–B line method, in which machines turn at the end of each section and continue operations on the next. This approach explicitly accounts for each machine’s turning time, reflecting its distinct maneuverability characteristics and associated factors such as fuel consumption.

(3) At least one agricultural machine must start from position 1, initiating operations from the first ridge of the farmland to ensure complete coverage. Other agricultural machines have the flexibility to start from any of the total number of ridges available.

### Adaptation function

In multi-machine collaborative task allocation, the distribution of tasks directly affects the efficiency of agricultural machinery and overall farm operational costs. This study adopts total collaborative operation time and fuel consumption as the primary optimization metrics to maximize agricultural productivity. This approach ensures that the task allocation scheme optimized by the algorithm satisfies the operational requirements of unmanned farms.

In this paper, we propose an efficient task allocation management mechanism along with its corresponding algorithm, specifically designed for cooperative task allocation across multiple farm fields and multiple machines. The model can be succinctly described as assigning the operational tasks of m total fields to n agricultural machines based on operational efficiency and economic benefits. The representation of the field is formalized in Eq (1).

$$M=[\begin{array}{c} 1,2,3,\ldots ,m \end{array}],m\in \{\begin{array}{c} 200,250,300,350 \end{array}\}$$ (1)

All agricultural machines have the same basic attributes except for speed, and agricultural machines are represented as shown in Eq (2).

$$N=[\begin{array}{c} n_{1},n_{2},n_{3},\cdots ,n_{N} \end{array}]$$ (2)

Due to the different speeds of each farm machine, the operational efficiency of different farm machines is different, and the economic efficiency of the operation is also different. The corresponding speeds of different agricultural machines are shown in Eq (3).

$$S=[\begin{array}{c} s_{1},s_{2},s_{3},\cdots ,s_{n} \end{array}].$$ (3)

For this study, the speed is abstracted as an integer, which denotes the speed of agricultural machine No. 1, meaning the time it takes for the agricultural machine to work one field of mounds. Assumption S=[5,6,7,8,9] shows that there are five agricultural machines, in which agricultural machine No. 1 will take five units of time to complete the operation of one field of mounds, agricultural machine No. 2 will take six minutes, and so on. So, the smaller the number representing speed, the faster the farm machine is, and vice versa. The larger the number, the slower the machine. The distribution scheme is shown in Eq (4).

$$T=[\begin{array}{c} t_{1},t_{2},t_{3},\ldots ,t_{n} \end{array}].$$ (4)

Here, *t*_1_ represents the turning time for Farm Machine No. 1, denoting the unit time required for each turn. Assuming T=[2,3,6,4,5] indicates a total of five farm machines, Farm Machine No. 1 requires 2 minutes to turn, Farm Machine No. 2 requires 3 minutes, and so on.

The fuel consumption of each piece of agricultural machinery is expressed as shown in Eq (5).

$$F=[\begin{array}{c} f_{1},f_{2},f_{3},\ldots ,f_{n} \end{array}].$$ (5)

Here, *f*_1_ denotes the fuel consumption rate of Farm Machine No. 1, corresponding to *f*_1_ liters per minute. Let F=[0.15,0.1,0.2,0.1,0.1] represent the total number of farm machines, which is five. Specifically, Farm Machine No. 1 consumes 0.15 liters of fuel per minute, Farm Machine No. 2 consumes 0.1 liters per minute, and so forth for the remaining machines.

The distribution scheme is shown in Eq (6).

$$U=[\begin{array}{c} u_{1},u_{2},u_{3},\cdots ,u_{n} \end{array}]\left(u\in [1,m]\right)$$ (6)

U stores the allocation results, and the numbers in U indicate the starting field tasks assigned to different agricultural machines. The number of columns corresponds to the number of agricultural machines. Suppose U is [47,1,138,178,225], where *u*_1_ is 47, indicating that the starting row of agricultural machine No. 1 is 47th. The operation task is from 47 to 137 rows. *u*_2_ is 1, which means that the starting row of agricultural machine No. 2 is the first row. The task is from 1 to 46, and so on.

The operation of the agricultural machinery in the farmland is represented by Eq (7).

$$A={\left[\begin{array}{c} 0,0,0,\ldots ,0 \end{array}\right]}^{T}$$ (7)

The number of rows of A represents the number of rows in the farmland, with 0 indicating that the row has not been operated and 1 indicating that the operation of the row has been completed. According to the task allocation result, the starting number of rows for each agricultural machinery is determined, and if the current monopoly operation task is not completed, the corresponding agricultural machinery speed s is reduced by 1, and the synergy time is added by 1. Simultaneously, the cumulative fuel consumption of each agricultural machine is updated, as defined in Eq (8).

$$Total\_fuel=Total\_fuel+f(n_{i})$$ (8)

Here, Total_fuel denotes the total fuel consumption, and *f*(*n*_*i*_) represents the fuel consumption of the i-th agricultural machine.

When s is 0, the current single-machine operation task has been completed. Subsequently, *A*(*u*) is updated from 0 to 1, and the task serial number u is incremented by 1. At the same time, the corresponding agricultural machine’s speed is reset, and the associated turning time is accumulated. The specific formula is shown in Eq (9).

$$A(u)=\left\{ \begin{array}{c} 1,s=0\\ 0,s\geq 0 \end{array}\right.$$ (9)

Finally, to determine whether all the agricultural machine operation tasks are completed, the task completion is determined by summing the array A to the total number of field mounds. Moreover, the coordinated operation time of the current task assignment result is recorded in TIME, as shown in Eq (10).

$$Sum(A)=\sum_{i=1}^{n}a_{i}$$ (10)

This study evaluates the quality of task allocation results based on collaborative operation time and fuel consumption. Specifically, the effectiveness of a given task allocation is assessed according to the time required under specified conditions, including the number of agricultural machines, their operational speeds, turning times, and fuel consumption rates. Shorter task completion times indicate higher allocation efficiency, which reduces operational costs while substantially enhancing overall productivity. Similarly, lower fuel consumption reduces energy expenses and mitigates environmental impact, improving operations’ economic efficiency and sustainability. The analytical function of the multi-machine coordination model is presented in Eq (11).

f(x)=Min(α·time+β·Total_fuel) (11)

**Fig 1:**
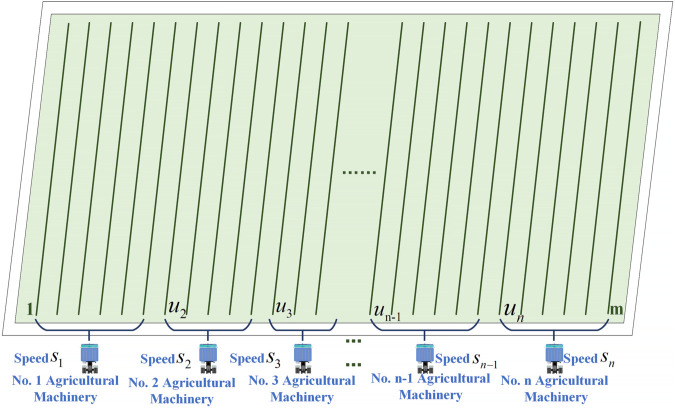
Tasking model. This figure visually presents the task allocation model.

Having a reasonable task allocation scheme in the context of multi-machine collaboration on the same farmland not only ensures that farm machinery is used efficiently, but also lowers costs, increases the actual economic value of the farmland, and significantly improves the efficiency of multi-machine collaboration. The following figure visually depicts the task assignment model (Fig 1).

Time represents the collaborative operation time, Total_fuel denotes the total fuel consumption, and *α* and *β* are their respective weighting coefficients, which quantify the relative importance of collaborative time and fuel consumption within the overall optimization objective. In this study, collaborative time and fuel consumption are considered equally important, each assigned a weight of 0.5. By balancing these two objectives, the optimization model prevents excessive bias toward either metric.

**Fig 2:**
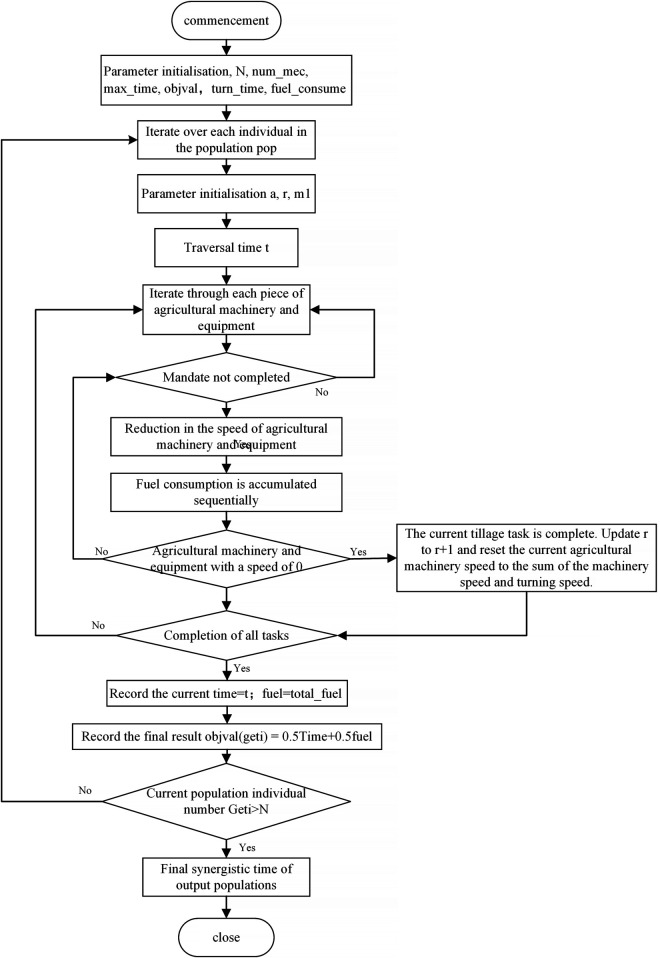
Flowchart of the fitness function. This figure illustrates the overall computational process of the fitness function.

The fundamental principle of the fitness function is to uniformly quantify the entire operation process using a global time variable t. At each time step, the status of all agricultural machines is evaluated and updated synchronously. If the ridge assigned to a machine remains unfinished, the remaining operation time is decremented, and fuel consumption accumulates. When a machine’s remaining time reaches zero, the corresponding ridge is marked as completed, and the machine is reassigned to the next ridge, with its remaining time reset to the sum of the operation and turning times. This cycle repeats until all ridges are completed. At this point, t represents the total collaborative operation time, and Total_fuel denotes the total fuel consumption. Finally, a fitness value is obtained by weighting and combining these two metrics, enabling a quantitative evaluation of the task allocation scheme at the multi-objective optimization level. The corresponding flowchart is shown in Fig 2.

### Evaluation indicators

#### Collaborative algorithm task efficiency improvement rate.

When evaluating task assignment efficiency, task completion time is crucial. It denotes the total duration spanning from initiating task allocation to completing tasks by all farm machinery involved. A shorter task completion time signifies more effective task allocation and higher efficiency in the collaborative efforts of agricultural machines.

To gauge the enhancement in operational efficiency of cooperative algorithms, we first compute the time differential between each algorithm’s cooperative operation and the CCEVSOA algorithm under various constraints. This difference quantifies the improvement of the CCEVSOA algorithm in terms of time. We can quantify this improvement in efficiency using Eq (12) to calculate the percentage improvement, thereby quantifying the degree to which cooperative operation efficiency has been enhanced.

$$\eta_{imp}=\frac{\left(Time_{orig}-Time_{CCEVSOA}\right)}{Time_{orig}}\times 100\%$$ (12)

Where $\eta_{imp}$ denotes the percentage increase in the efficiency of multi-computer synergy,*Time*_*orig*_ denotes the synergy time obtained by the original algorithm, and $Time_{CCEVSOA}$ denotes the synergy time obtained by the algorithm CCEVSOA.

#### Agricultural machinery load fairness calculation.

Load fairness evaluation metrics assess the balance of task distribution within multi-agricultural machinery systems by quantifying differences in workloads—such as operating time and travel distance—across different machines. Higher load balance indicates a fairer task allocation, preventing scenarios in which some machines remain idle while others operate under sustained high loads. This, in turn, enhances overall system stability and resource utilization.

In this study, load fairness is quantified using the Jain fairness index, which effectively reflects the uniformity of load distribution in multi-machine systems. The total working time of each machine is first calculated, encompassing the operation time for the first furrow, subsequent furrows, and all turning maneuvers. Based on the resulting set of working times, the load fairness index for the task allocation scheme is computed according to Eq (13).

$$Loads(n_{1},n_{2},n_{3},...,n_{n})=\frac{{\left(\sum_{i=1}^{n}{time}_{i}\right)}^{2}}{n\cdot \sum_{i=1}^{n}{time}_{i}^{2}}$$ (13)

Here, *time*_*i*_ represents the load of the i-th agricultural machine, measured in terms of operating time; n denotes the number of objects; and *Loads* corresponds to the load fairness index of the agricultural machines. The closer *Loads* is to 1, the more balanced the task allocation. This metric serves as a key basis for comprehensively evaluating the rationality of task allocation schemes and the overall collaborative efficiency of the system.

## Theory and calculations

In this paper, a novel CCEVSOA algorithm is proposed for the efficient assignment of multiple agricultural machinery operation tasks. The algorithm introduces a new chaos operator to ensure initial solutions are evenly distributed across the search space, enhancing population diversity. Additionally, a novel Cauchy variation operator is designed to diversify the population further, thereby improving global search capabilities and expanding the exploration of the search space to prevent local optima. Furthermore, an elitist evolution strategy is introduced to retain high-performing individuals and extend the search space for finding superior solutions.

Through the application of CCEVSOA, efficient and rational task allocation among multiple agricultural machines within the same farmland is achieved, effectively enhancing fleet operational efficiency and reducing costs.

### Snake optimization algorithm

SO is a novel nature-inspired heuristic algorithm introduced by Professors Hashim, F. A. and Hussien, A. G. in 2022 [33]. Aiming for efficiency and speed, this algorithm mimics snakes’ foraging and reproduction behaviors. It simulates various behavioral patterns influenced by environmental conditions such as temperature and food availability to optimize finding optimal values.

Inspired by snake mating behaviors, SO divides its search process into exploration and exploitation. During exploration, when food is scarce, the snake population explores to find food. In contrast, when food is abundant during exploitation, the population engages in foraging, fighting, and mating behaviors based on temperature conditions. Higher temperatures prioritize food foraging, while lower temperatures induce fighting or mating. Mating produces new individuals that replace the least-fit males and females.

Building upon the Snake Optimization algorithm, this paper proposes CCEVSOA. CCEVSOA integrates a new chaos operator, a Cauchy variational operator, and an elite evolutionary strategy. The new chaos operator enhances the uniformity of initial multi-machine collaborative task allocation schemes, thereby improving algorithm convergence speed. The Cauchy variational operator enhances algorithmic exploration capabilities to mitigate rapid convergence to local optima. The elite evolutionary strategy also ensures optimal individual retention across generations, enhancing overall population adaptability and performance.

### Initialization of population

The initial task allocation scheme determines the time required for multi-machine cooperative operations. An inappropriate initial position can lead to the overloading or idling of agricultural machinery, ultimately reducing operational efficiency. Ensuring a rational initial task allocation can effectively mitigate these issues, leading to reduced cooperative operation time. Chaotic operators, known for their nonlinearity, robustness, and enhanced global search capabilities, offer a means to improve solution quality. Applying chaotic sequences in initial task allocation enhances solution quality, thereby preventing the overloading or idling of agricultural machinery.

In this context, the CCEVSOA algorithm introduces a novel chaos operator during population initialization, designed to achieve a more rational task allocation scheme for multi-machine collaboration. The new operator increases the randomness of the population distribution, thereby accelerating the algorithm’s convergence speed and improving its overall efficiency.

**Fig 3:**
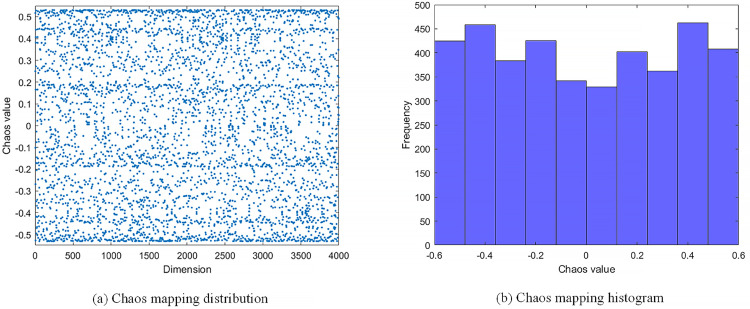
Chaos distribution map.

The chaotic operator generates sequences characterized by enhanced randomness and uncertainty, contributing to improved algorithm performance. These sequences exhibit strong randomness and effective traversal properties, ensuring a more thorough solution for space exploration. The chaotic mapping distribution and histogram are illustrated in Fig 3 (a) and (b). These figures demonstrate that the proposed chaotic operator achieves a uniform distribution, further validating its effectiveness.

By ensuring that the initial task allocation scheme is balanced and efficient, this approach establishes a solid foundation for subsequent optimization steps within the CCEVSOA algorithm, ultimately enhancing the overall performance of multi-machine collaborative task allocation.

The expression for chaotic sequence generation is shown in Eq (14).

$$z(n+1)=4\times z(n)\times (1-z(n))\left\{n\in N^{*}\right\}$$ (14)

Where z(n)∈(0,1) uses the stochastic nature of chaotic sequences, maps them to the range of values of the variables to be optimized, and searches for the initial allocation scheme through the chaotic properties of chaotic sequences. The specific steps are shown below.

1) The D-dimensional space has F individuals, and a D-dimensional vector is randomly generated as the initial individual, as shown in Eq (15)

$$R=\{\begin{array}{cc} r_{1},r_{2},r_{3},r_{4} & \cdots \end{array}\}(\begin{array}{c} r_{i}\in \left(0,1\right),1\leq i\leq d \end{array})$$ (15)

2) Generate the remaining N-1 individuals by performing N-1 iterations on R via Eq (15).

3) Map the above obtained chaotic variables into the search space of solutions according to Eq (16).

$$\left\lfloor x_{id}=\frac{\left(1+r_{id})\times (x_{u}-x_{l}\right)}{3}\right\rfloor$$ (16)

In the above equation, *x*_*id*_ denotes the position of individual i in the d-dimension, *r*_*id*_ denotes the value of the d-dimension obtained by individual i according to Eq (16). *x*_*u*_ denotes the upper boundary of the problem to be solved, and denotes the lower boundary.

4) Randomly generate a positive integer pos value smaller than the dimension, and set the number corresponding to the position of this pos value to 1, to ensure that there is a number of 1 in the result of the task assignment, so as to ensure that there is a farm machine from the starting position to start the operation, which can realize the full path coverage of the farmland.

In CCEVSOA, an individual’s adaptation degree is defined by the time required to determine task allocation, which depends on the number and speed of farm machines. A lower adaptation degree signifies quicker task completion by the group of farm machines under this allocation scheme, indicating higher efficiency in multi-machine cooperative operations within the same farmland. The method for calculating individual adaptation degrees is detailed in the Adaptation Function section of Materials and Methods. Within CCEVSOA, the population is categorized into male and female subsets. This study assumes that males constitute 50% of the total population, with females comprising the remainder, as represented in Eq (17) and Eq (18). Subsequently, these subsets are independently processed, and after adaptation calculations, the individual with the maximum adaptation is identified through comparison.

$$F_{m}=\left\lfloor \frac{F}{2}\right\rfloor$$ (17)

$$F_{f}=F-F_{m}$$ (18)

Based on the equation above, represents the number of multi-machine cooperative task allocation results in the same farmland, *F*_*m*_ represents the number of males, and *F*_*f*_ represents the number of females.

### Corsi variation in CCEVSOA populations

Enhancing the algorithms’ search space capability in collaborative multi-machine operations is crucial for achieving high-quality task allocation optimization results. The Cauchy variation operator increases the diversity and search space of the population by randomly sampling variations from the Cauchy distribution and applying these variations to the individuals within the population. Additionally, the Cauchy variation operator features a smaller peak at the origin and a longer tail at the extremes, exhibiting the long-tailed property characteristic of the Cauchy distribution. Leveraging this property, the CCEVSOA is more likely to escape local optima and effectively approach global optima, thus enhancing its robustness against outliers. In the context of multi-machine cooperative operations, integrating the Cauchy variation operator can significantly boost the algorithm’s exploration capability, thereby facilitating the discovery of superior solutions in complex task allocation scenarios. This enhancement contributes to more effective task distribution and optimizes overall operational efficiency and resource utilization. By broadening the algorithm’s search capabilities, the Cauchy variation operator helps ensure that the solutions are more robust and better suited to the intricacies of the task allocation problem.

The Cauchy variation operator is shown in Eq (19).

rCauchy=tan((X−0.5)×π) (19)

X in Eq (19) is the random generation of a D-dimensional vector $X=\left\{x_{1},x_{2},x_{3},x_{4}\cdots \right\},x_{i}\in \left(0,1\right),1\leq i\leq d$ in the D-dimensional vector. After discovering the current optimal solution, this paper uses the updated formula shown in Eq (20) to mutate the current optimal solution.

In order to maximize the advantages of the Cauchy variational operator, after the SO completes the population division, the optimal solution of the obtained population is mutated, and the final solution is determined by comparing the mutated solution with the current optimal solution. The following steps introduce the Cauchy variation operator.

$$x_{newbest}=\left\lceil x_{best}+\left|rCauchy\right|\times x_{best}\right\rceil$$ (20)

(1) After finding the current optimal solution, this paper uses the update formulation shown in Eq (20) to mutate the current optimal solution.

Where *x*_*newbest*_ is the solution after mutation, *x*_*best*_ is the current optimal solution, and *rCauchy* is the Cauchy mutation operator.

(2) After the Cauchy variation is completed, a boundary check is performed on the result of this task assignment to ensure that it is within the range of the solution, after which the fitness is computed from the fitness function based on the result of the variant assignment.

(3) The fitness of the current optimal solution is compared with the fitness of the mutated solution, and the solution with small fitness is retained as the optimal solution for the generation.

### CCEVSOA’s elite evolutionary strategy

In the context of multi-machine cooperative operations, rational allocating tasks is essential for enhancing operational efficiency and reducing costs. However, traditional optimization algorithms often converge to local optima when faced with complex multi-machine collaborative problems, resulting in unreasonable task allocation schemes that adversely affect overall operational efficiency.

To address this issue, the elite evolution strategy mitigates the risk of losing high-performing individuals by retaining some of the best-performing solutions in each generation. This approach enhances the algorithm’s stability and provides higher-quality solutions for subsequent evolutionary processes, ensuring that the algorithm can continue to converge toward superior solutions. Consequently, it facilitates the attainment of a reasonable task allocation scheme, thereby improving resource utilization, reducing costs, and promoting agricultural economic development.

(1) Set an elite pool with the number of rows as the number of iterations and the number of columns as the number of dimensions for retaining the best individuals in the population in all generations.

(2) Starting from the second generation, store the optimal solution of each generation in the elite pool and randomly select an individual from the elite pool.

(3) Remove the individual from the elite pool and place it into the male and female populations of the next population, respectively, and at the same time, place one of the best individuals from the current iteration into the next generation for co-evolution.

### Steps for multi-machine task assignment using CCEVSOA

The operational process of the CCEVSOA-based multi-agricultural task assignment can be categorized into the following steps.

**Step 1:** Algorithm initialization, given the relevant parameters. The number of individuals in the population F, the maximum number of iterations T, the number of problems D, the upper boundary *x*_*l*_, the lower boundary *x*_*u*_, the number of ridges number field, the speed of agricultural machines speed and the number of agricultural machines number machinery.

**Step 2:** Chaos initialization, according to Eq (14) and Eq (15), the population is initialized using chaos operators to improve the randomness of task allocation and to ensure that one agricultural machine starts plowing from the first ridge so that the allocation results are in line with the multi-machine collaborative scenario.

**Step 3:** The population was divided into two groups, females and males, according to Eq (17) and Eq (18). Population fitness was calculated, optimal individuals were found, and values and positions were recorded.

**Step 4:** Calculate the quantity of food Q and the temperature T.

**Step 5:** A judgment is made on Q. If Q is less than a given food threshold of 0.25, then the current global optimal fitness is found. If Q is greater than the food threshold, judge the temperature T.

**Step 6:** A judgment is made on T. If T is greater than a given temperature threshold of 0.6, then the population will enter exploitation mode, updating individual locations based on food status. If T is less than the temperature threshold, a judgment is made on the population density $\rho_{k}$

**Step 7:** If $\rho_{k}>0.6$, the population enters the fighting phase, where individuals are positionally updated based on their fighting ability. If $\rho_{k}\leq 0.6$, the population enters the mating phase, where positions are updated based on temperature and the population’s ability to reproduce, and eggs are laid.

**Step 8:** If the eggs hatch, the worst-adapted males and females in the population are replaced. Moreover, the best fitness in the world has been updated.

**Step 9:** When the number of iterations exceeds 2, the global best fitness and allocation results are put into the elite pool. An individual is randomly selected from the elite pool to be placed into the next generation of male and female populations. One of the best individuals from the current iterative process is placed into the next generation for co-evolution.

**Step 10:** After obtaining the current optimal solution, the current global optimal solution is subjected to Kersey variation through Eq (19) and Eq (20), and the updated result is compared with the previous global optimal solution to obtain the optimal solution.

**Step 11:** Evaluate the current iteration count. If it remains below the total iteration threshold T, return to Step 3. Otherwise, proceed to output the final task assignment results for the multi-agricultural machine operation.

**Fig 4:**
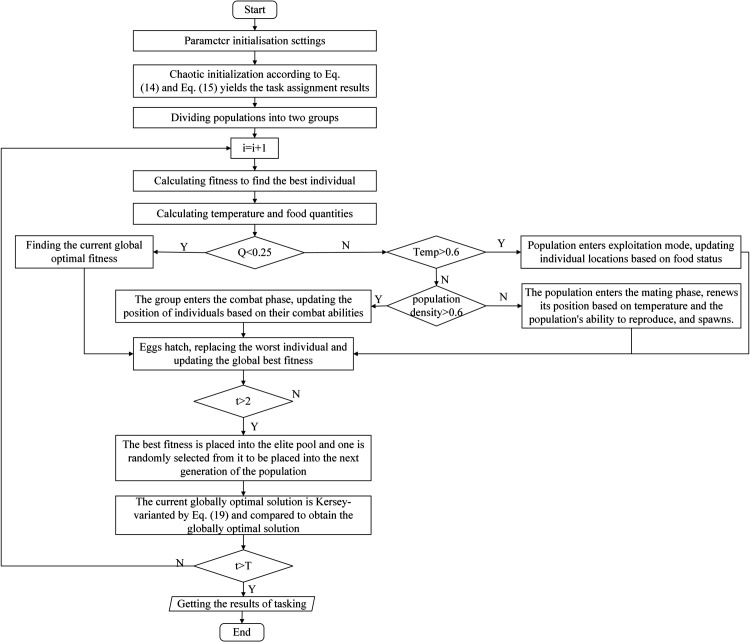
Flow chart of CCEVSOA.

The flowchart of CCEVSOA is shown in Fig 4:

### CCEVSOA complexity analysis

The main sources of complexity in the CCEVSOA proposed in this paper are SO and chaos operator. The complexity of SO is mainly affected by the number of iterations, spatial dimensions, the number of tasks to be processed and the number of individuals, which lead to an increase in the algorithm’s computational complexity. The complexity of SO in CCEVSOA can be expressed as

$$CCEVSOA_{so}=d\times F\times T\times s$$ (21)

In the above equation, F denotes the number of individuals included in the population, T is the maximum number of iterations, d is the dimensionality of the solution space, and s denotes the total number of tasks assigned.

The chaotic sequence of initialized population individuals and the enhanced randomness of the population operation both exhibit the complexity of the chaotic operator, so the time complexity of the chaotic operator can be expressed as

$$CCEVSOA_{Chaotic}=\log_{2}(K+s)$$ (22)

Consequently, the overall computational complexity of CCEVSOA can be expressed as follows.

$$CCEVSOA_{Total}=s\times F\times T\times d+\log_{2}(F+s)$$ (23)

The time complexity of the SO algorithm is mainly affected by the initialization of the population, position update of the female and male populations and iterative computation during the algorithm operation. The time complexity of female and male populations is calculated as shown in Eq (24) and Eq (25).

$$SO_{m}=s\times {F_{m}}^{2}\times T\times d$$ (24)

$$SO_{f}=s\times {F_{f}}^{2}\times T\times d$$ (25)

In the above equation, the number of monograms denotes the total number of tasks assigned to the task, T is the maximum number of iterations, and d is the dimensionality of the solution space. Moreover, it denotes the number of male and female individuals in the population, respectively.

SO overall time complexity is

$$SO_{Total}=\frac{1}{2}\times s\times F^{2}\times T\times d$$ (26)

In order to facilitate the comparison of the complexity of CCEVSOA and SO, the formulas are simplified by deleting the lower order terms, constant terms and coefficients in the formulas and utilizing the large O representation.

$$CCEVSOA=O(s\times F\times T\times d+\log_{2}(F+s))$$ (27)

$$SO=O(s\times F^{2}\times T\times d)$$ (28)

For Eq (27), the growth rate of the logarithmic function is negligible relative to F and T. Also, since both algorithms have the same parameter settings, simplifying Eq (27) and Eq (28) yields.

CCEVSOA=O(F) (29)

$$SO=O(F^{2})$$ (30)

Comparing Eq (29) and Eq (30), it can be seen that the CCEVSOA complexity is lower than SO, i.e., CCEVSOA will not increase the running cost of the same task, and the running time will be shorter.

## Results

A series of simulation experiments were conducted to verify the effectiveness of CCEVSOA, which is proposed in this paper, in improving the operational efficiency of agricultural machines. Moreover, CCEVSOA was compared with SO, CSA, GA, WOA, etc. The experimental results described in this paper are the average of the effects of 100 experiments. The simulation experiments consisted of comparing the efficiency of fleet operations. Different numbers of farm machines, farm speeds, and field mounds were used to verify the usability and robustness of CCEVSOA. In addition, all simulations were performed on a computer equipped with i5-8250U CPU @ 1.60GHz and the fitness function 3.3 was followed in the algorithm.

In order to better compare the performance of different algorithms in the process of task allocation of agricultural machinery fleet, the same parameters are used to ensure the objectivity and fairness of the simulation experiment. In the simulation experiment, the population size of all algorithms is set to 30, and the maximum number of iterations is set to 300. The detailed parameter settings of the six algorithms are shown in Table 1.

**Table 1:** Algorithm detailed parameter settings.

Algorithm name	Basic parameters of the algorithm
CCEVSOA	s=30, T=300, c1=0.5, c2=0.05, c3=2
SO	s=30, T=300, c1=0.5, c2=0.5, c3=2 [34]
GA	s=30, T=300, Pm=0.01, Pc=0.8 [10]
CSA	s=30, T=300 [35]
WOA	s=30, T=300, lb=0, ub=300 [36]
IBES	s=30, T=300 [37]

Table notes: “s” is the population size, “T” is the maximum number of iterations, “c1–c3” are algorithm-specific coefficients, “Pm” is mutation probability, “Pc” is crossover probability, and “lb/ub” are lower/upper bounds of the search space.

The simulation constraints for different numbers of agricultural machines, machine speeds, and field ridge counts are summarized in Table 2. Constraints 1 and 2 constitute Simulation Experiment 1, where machine speed is the variable for evaluating the algorithm’s generality. Constraints 1 and 3 define Simulation Experiment 2, with machine quantity and speed as variables, to assess the algorithm’s effectiveness in multi-machine task allocation under varying machine numbers. Constraints 1 and 4 form Simulation Experiment 3, in which the number of field ridges is varied to evaluate the algorithm’s multi-machine coordination performance for different total task allocations. Constraint 5 serves as the test condition for large-scale field scenarios, validating the algorithm’s robustness and scalability in high-complexity, large-scale task allocation problems and assessing its practical applicability in real agricultural production environments. Table 3 presents the optimized results of collaborative operation time, total fuel consumption, fitness values, load fairness, and task allocation for the four simulation experiments using GA, CSA, WOA, SO, CCEVSOA, and IBES algorithms.

**Table 2:** Algorithm constraint settings.

Restrictive condition	Number of machines	Cornering speed	Fuel consumption per unit time	Agricultural Machinery Speed	Number of Ridges
1	5	2, 3, 6, 4, 5	0.15, 0.1, 0.2, 0.1, 0.1	5, 6, 7, 8, 9	300
2	5	2, 3, 6, 4, 5	0.15, 0.1, 0.2, 0.1, 0.1	6, 7, 8, 9, 10	300
3	6	2, 3, 6, 4, 2, 3	0.15, 0.1, 0.2, 0.1, 0.1, 0.15	5, 6, 7, 8, 9, 5	300
4	5	2, 3, 6, 4, 5	0.15, 0.1, 0.2, 0.1, 0.1	5, 6, 7, 8, 9	500
5	10	2, 3, 3, 2, 4	0.15, 0.1, 0.2, 0.1, 0.1	5, 6, 7, 8, 9	1000

Table notes: The constraint settings simulate varying working conditions for evaluating algorithm performance under different numbers of machines, operational speeds, and ridge counts.

**Table 3:** Comparison of GA, CSA, WOA, SO, CCEVSOA, and IBES under five conditions.

Condition	Algorithm	Collab. Time (min)	Fuel (L/min)	Fitness	Fairness	Task Allocation
1	GA	723	383.2	553.1	0.97415	1 97 212 170 249
1	CSA	709	364.25	536.625	0.94139	126 1 276 79 225
1	WOA	726	343.1	534.55	0.86476	52 166 156 242 1
1	SO	656	379.7	517.85	0.98295	156 83 1 250 36
1	**CCEVSOA**	**620**	**399.7**	**509.85**	**0.99995**	**213 1 69 117 169**
1	IBES	717	338	527.5	0.84535	8 221 1 161 110
2	GA	917	374.1	645.55	0.74383	10 209 193 124 1
2	CSA	750	425.5	587.75	0.9893	45 129 261 203 1
2	WOA	750	431.1	590.55	0.98445	157 1 72 115 251
2	SO	745	404.8	574.9	0.9496	1 94 168 194 251
2	**CCEVSOA**	**687**	**436.2**	**561.6**	**0.9992**	**211 1 168 116 71**
2	IBES	777	384.1	580.55	0.882	1 232 87 179 133
3	GA	609	356.6	482.8	0.87155	1 206 274 155 100 84
3	CSA	537	370.9	453.95	0.89835	226 42 101 1 135 184
3	WOA	525	373.05	449.025	0.90635	1 73 267 131 166 201
3	SO	597	320.6	458.8	0.77511	141 75 74 55 1 226
3	**CCEVSOA**	**537**	**325.5**	**431.25**	**0.94558**	**224 164 1 70 115 32**
3	IBES	527	381.1	454.05	0.91173	137 13 260 1 212 71
4	GA	1451	644.05	1047.525	0.83337	97 416 362 1 258
4	CSA	1132	655.3	893.65	0.94404	222 384 146 56 1
4	WOA	1088	649.7	868.85	0.9636	146 385 79 294 1
4	SO	1073	666.25	869.625	0.98846	177 1 418 330 120
4	**CCEVSOA**	**1041**	**661.65**	**851.325**	**0.99958**	**352 1 116 266 192**
4	IBES	1045	667.15	856.075	0.9989	81 230 421 1 346
5	GA	1667	1356.95	1511.975	0.80536	945 199 462 661 386 122 513 788 366 1
5	CSA	1477	1159.2	1318.1	0.77595	32 561 1000 358 1 709 199 915 470 448
5	WOA	1468	1181.05	1324.525	0.81474	375 760 752 302 229 804 1 161 556 703
5	SO	1383	1133.5	1258.25	0.85384	718 489 1 23 428 232 847 1 581 129
5	**CCEVSOA**	**1177**	**1248.9**	**1212.95**	**0.92608**	**652 534 930 1 266 79 799 247 417 332**
5	IBES	1240	1257.85	1248.925	0.86505	39 245 862 590 507 685 369 877 1 194

The table presents a comparison of six algorithms under five conditions, showing collaboration time, fuel consumption, fitness, fairness, and task allocation. Bold values indicate the best-performing algorithm under each condition based on collaboration time.

**Fig 5:**
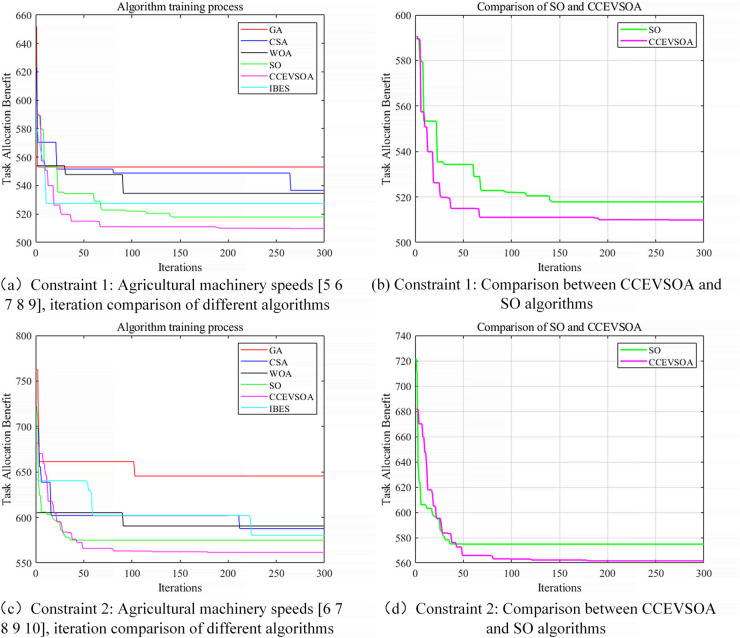
Iterative curves for algorithm comparison under different agricultural machinery speed constraints.

Fig 5(a)–5(d) compare the cooperative operation performance and task allocation results of IBES, GA, CSA, WOA, SO, and the proposed CCEVSOA algorithm under different agricultural machinery speeds. The results clearly demonstrate that, across various speed conditions, the CCEVSOA algorithm consistently achieves the shortest collaborative operation time and the lowest fitness value, indicating superior collaborative efficiency. Furthermore, its load fairness index is closest to 1, reflecting excellent load balancing in the task allocation results.

Taking Fig 5(a) as an example, when the speeds of five agricultural machines are [5, 6, 7, 8, 9], the collaborative operation times obtained by GA, CSA, WOA, IBES, and SO are 723, 709, 726, 717, and 656 minutes, respectively. By contrast, the CCEVSOA algorithm reduces the collaborative operation time to 620 minutes, achieving time savings of 103, 89, 106, 97, and 36 minutes compared with the aforementioned algorithms, corresponding to efficiency improvements of 14.25%, 12.55%, 14.6%, 13.53%, and 5.5%, respectively. Additionally, CCEVSOA attains the lowest overall fitness value among all algorithms, highlighting its significant advantage in optimizing operation time and fuel consumption simultaneously. This balance between efficiency and energy consumption enhances the system’s economic viability and practical applicability. Its superior load-balancing capability also mitigates single-unit overload, improving long-term operational stability. Specifically, Fig 5(b) provides a direct comparison between CCEVSOA and the SO algorithm, demonstrating that CCEVSOA outperforms SO in both convergence speed and solution quality, validating the effectiveness of its enhanced optimization strategy.

From an algorithmic perspective, GAS exhibits limited global exploration capabilities when addressing such problems and is prone to convergence to local optima. Although CSA, WOA, and IBES outperform GA, they still show limited effectiveness in locating global optima. The SO algorithm performs relatively better but is susceptible to the initial population and is particularly prone to premature convergence in complex, high-dimensional search spaces.

The proposed CCEVSOA algorithm effectively overcomes these limitations through three key enhancements. First, a newly designed chaotic operator is introduced for population initialization, improving population diversity and accelerating convergence. Second, the Cauchy mutation operator expands the search range within the solution space, enhancing the ability to escape local extrema. Finally, an elite evolution strategy is adopted to optimize the individual elimination mechanism, further strengthening global search performance. As illustrated in Fig 5(a) and 5(b), CCEVSOA converges substantially faster than the comparison algorithms and produces higher-quality final solutions, indicating that these improvements synergistically enhance the algorithm’s overall optimization performance.

**Fig 6:**
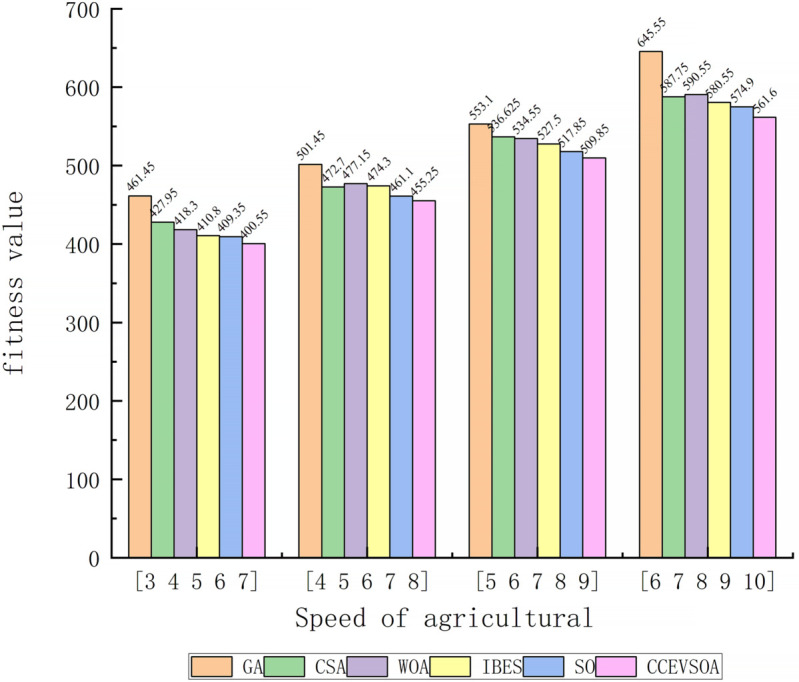
Bar chart comparing algorithms at different agricultural machinery speeds.

Fig 5(c) shows that when the speeds of the five agricultural machines are [6, 7, 8, 9, 10], the collaborative operation efficiency optimized by CCEVSOA improves by 25.08%, 8.4%, 8.4%, 11.58%, and 7.79% compared with GA, CSA, WOA, IBES, and SO, respectively. Fig 6 presents a bar chart comparing task allocation results across the optimization algorithms under varying agricultural machinery speeds. It is evident that CCEVSOA consistently outperforms the other algorithms across multiple speed combinations. Analysis of Figs 5 and 6 indicates that the proposed CCEVSOA algorithm exhibits both universality and robustness in addressing multi-machine cooperative task allocation problems under different speed conditions. The optimized CCEVSOA algorithm achieves lower collaborative operation times and fitness values than GA, CSA, WOA, IBES, and SO under various speed constraints. These comparative results demonstrate that the improved strategies effectively enhance the algorithm’s optimization capability, enabling it to escape local optima and achieve near-optimal task allocation outcomes.

**Fig 7:**
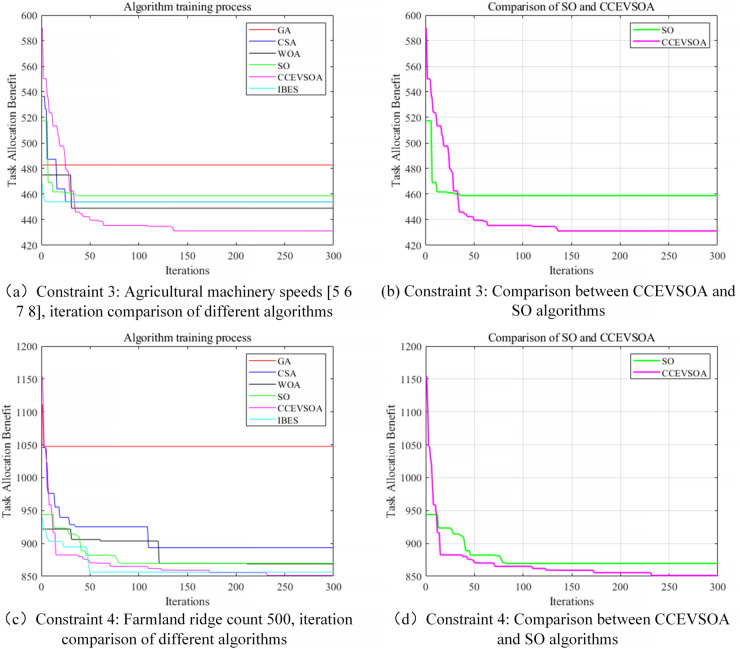
Iteration curves comparing algorithms under different constraints of agricultural machinery quantity and field ridge count.

**Fig 8:**
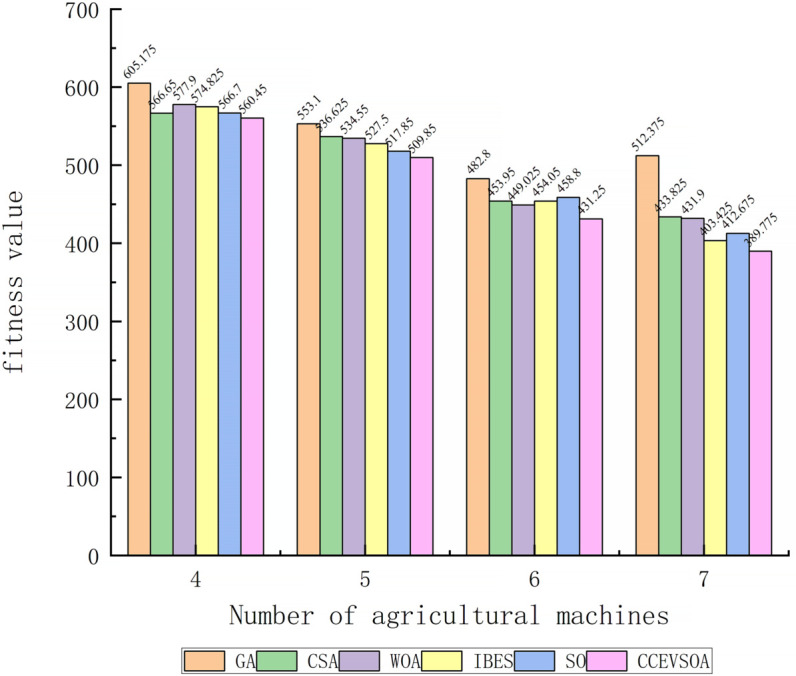
Bar chart comparing algorithms under different numbers of agricultural machines.

**Fig 9:**
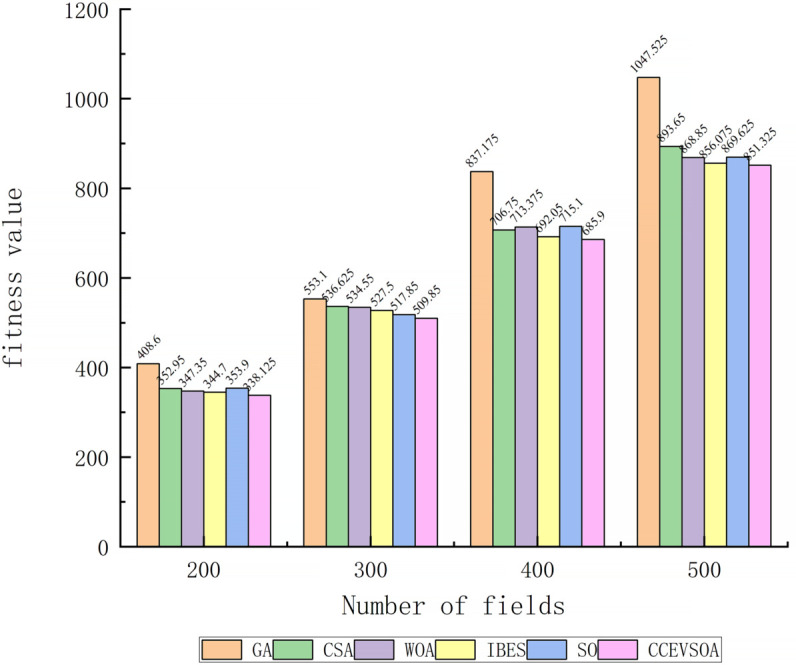
Bar chart comparing algorithms under different number of farmland ridges.

Fig 7(a) illustrates the iterative curves of collaborative operation time for GA, CSA, WOA, IBES, SO, and the improved CCEVSOA algorithms under varying numbers of agricultural machines and speed constraints. The results indicate that, when the number of machines is six, CCEVSOA consistently achieves superior collaborative operation times in task allocation compared with GA, CSA, WOA, and SO. Specifically, when six machines with speeds [5, 6, 7, 8, 9, 10] execute tasks, CCEVSOA attains an optimal fitness value of 431.25, representing reductions of 51.55, 22.695, 17.77, 22.8, and 27 compared with the aforementioned algorithms, while achieving a load fairness index closest to 1. These results demonstrate that CCEVSOA effectively reduces overall collaborative operation time and exhibits superior resource allocation rationality and operational load balance, thereby significantly enhancing the overall efficiency and robustness of multi-agricultural machinery collaboration. Fig 8 compares the fitness values of all algorithms under different numbers of agricultural machines. Integrating the results from Figs 5(a), 7(a), and 8 reveals that CCEVSOA consistently performs superior optimization in multi-machine task allocation across varying machine quantities. Fig 7(c) presents the iterative curves of collaborative operation time for GA, CSA, WOA, IBES, SO, and CCEVSOA when the number of field ridges is 500. Under this constraint, the collaborative operation time achieved by CCEVSOA’s task allocation decreases by 410, 91, 47, 4, and 28 minutes compared with GA, CSA, WOA, IBES, and SO, respectively, corresponding to efficiency improvements of 28.3%, 8%, 4.3%, and 2.6%. Moreover, CCEVSOA attains a lower overall fitness value than the other algorithms, highlighting its advantage in comprehensively optimizing operation time and fuel consumption. This demonstrates the algorithm’s ability to enhance efficiency while controlling energy consumption. Fig 9 presents the fitness values of each algorithm under varying numbers of farmland ridges. It is evident that, even when the total task volume changes due to different ridge configurations, CCEVSOA maintains stable superiority in multi-machine task allocation, demonstrating consistently higher optimization performance and adaptability.

**Fig 10:**
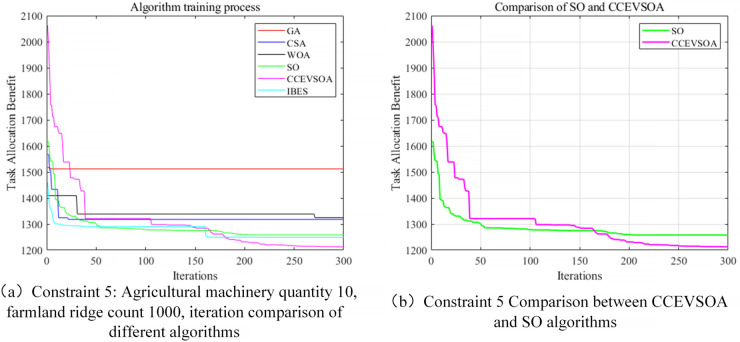
Iteration curves comparing algorithms under constraints of large-scale farmland ridge count and agricultural machinery quantity.

**Fig 11:**
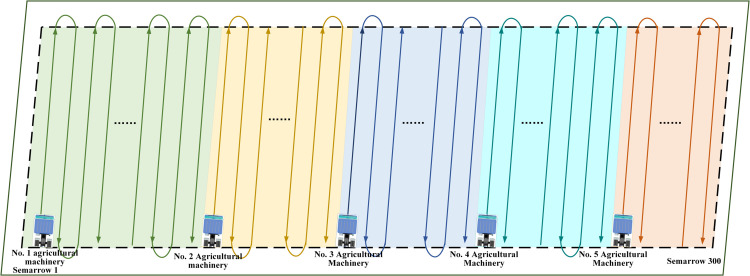
Schematic diagram of multi-aircraft collaborative task allocation.

Simulation experiments were conducted using 10 agricultural machines and 1,000 field ridges to validate the algorithm’s scalability in large-scale agricultural operations, with the results presented in Fig 10. The convergence curves indicate that, as the problem scale increases, algorithms such as GA, CSA, WOA, and SO exhibit significantly reduced convergence rates and achieve relatively long collaborative operation times in the final results, revealing specific performance bottlenecks. In contrast, CCEVSOA continues to converge rapidly to optimal solutions within fewer iterations. Compared with GA, CSA, WOA, IBES, and SO, the collaborative operation time is reduced by 490, 300, 291, 63, and 206 minutes, respectively, corresponding to improvements in collaborative efficiency of 29.40%, 20.31%, 19.82%, 5.08%, and 14.90%. Concurrently, CCEVSOA consistently maintains the lowest overall fitness value, indicating its capability to achieve a superior trade-off between task duration and fuel consumption in large-scale agricultural scenarios, demonstrating stronger comprehensive optimization performance. These results demonstrate that, even under large-scale task allocation conditions, CCEVSOA effectively preserves stable optimization performance, achieves good load balancing, and controls energy consumption, thereby ensuring the system’s efficiency and practical applicability. This further validates the scalability and generality of CCEVSOA in multi-machine collaborative task allocation problems. Fig 11 provides a schematic representation of the multi-machine cooperative task assignment framework. As illustrated, a regular parallelogram is selected as the demonstration area, and the field ridges are systematically allocated to five agricultural machines with varying speeds to facilitate multi-machine collaboration within the same field. The ridges are color-coded to indicate the distinct operational tasks assigned to each agricultural machine.

**Fig 12:**
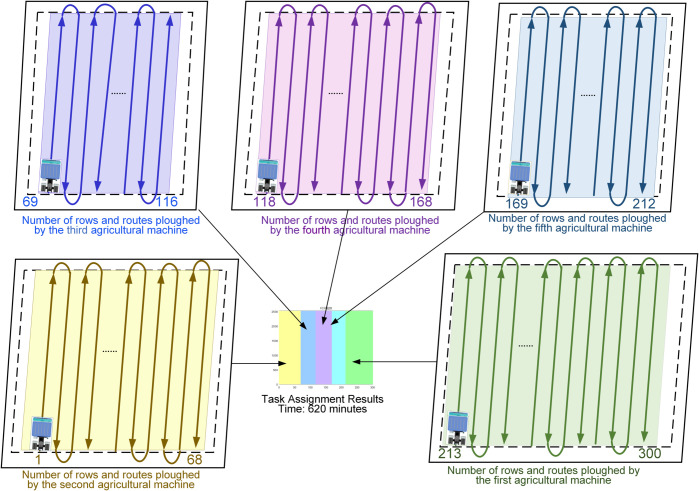
Optimization results for CCEVSOA with agricultural machinery speed [5 6 7 8 9] and ridge count below 300.

Fig 12 illustrates the task allocation results obtained using the CCEVSOA optimization algorithm under the following conditions: five agricultural machines, machine speeds of [5, 6, 7, 8, 9], turning times of [2, 3, 6, 4, 5], fuel consumption rates of [0.15, 0.1, 0.2, 0.1, 0.1], and 300 field ridges. The task allocation corresponds to agricultural machinery with 5, 6, 7, 8, and 9 speeds, respectively. In the figure, green represents machinery with speed 5, yellow represents speed 6, cyan represents speed 7, and purple and blue represent speeds 8 and 9, respectively.

According to the speed calculation formula, faster agricultural machines cover larger areas within the same period. Therefore, to achieve rational task allocation among multiple machines, faster machines should be assigned larger working areas, whereas slower machines should be assigned smaller areas. This allocation strategy ensures the shortest overall collaborative operation time. Conversely, unreasonable task allocation may result in some machines remaining idle while others are overloaded, increasing the total operation time.

Compared with GA, CSA, WOA, IBES, and SO, all algorithms except GA demonstrate superior optimization performance in multi-machine collaborative task allocation. Among these, CCEVSOA achieves the shortest collaborative operation time and significantly improves task allocation rationality by minimizing idle and overloaded machinery while reducing fuel consumption. This reflects its stronger comprehensive optimization capability in balancing operational efficiency and resource utilization. In a multi-machine cooperative context, reductions in task allocation coordination time and fuel consumption correspond to more rational task distribution, higher agricultural machinery utilization rates, and enhanced cooperative operation efficiency. These results confirm the effectiveness of CCEVSOA in optimizing multi-machine cooperative task allocation.

Furthermore, under identical field ridge configurations, CCEVSOA exhibits minimal sensitivity to variations in the number of machines or changes in machine speeds, demonstrating its general applicability. Consequently, employing CCEVSOA to optimize multi-machine task allocation within the same field results in lower economic costs, improved collaborative efficiency, and maximized economic benefits.

In summary, CCEVSOA achieves the shortest collaborative operation time in multi-machine task allocation compared with GA, CSA, WOA, IBES, and SO. Its fitness function comprehensively balances operational time and fuel consumption, optimizing temporal efficiency and energy utilization to deliver superior overall performance in multi-machine task allocation.

## Conclusion

This study addresses the multi-machine collaborative operations’ distinctive characteristics and requirements within the same farmland. Considering variations in agricultural machinery speeds, turning times, and fuel consumption, a novel task allocation model for agricultural machinery was developed. The model comprehensively captures the complex demands of multi-machine coordination and accurately reflects real-world task allocation conditions in unmanned farms. Based on this model, the proposed Chaotic Cauchy Elite Variation Snake Optimization Algorithm (CCEVSOA) implements an efficient task allocation strategy, substantially optimizing the multi-machine collaborative task allocation process by reducing collaborative operation time, lowering fuel consumption, and ensuring fairness in task load distribution. These improvements significantly enhance agricultural machinery operation efficiency and task allocation precision, thereby maximizing resource utilization, reducing operational costs, and supporting the sustainable development of the agricultural economy. The effectiveness of CCEVSOA arises from the integration of multiple optimization mechanisms. A novel chaotic operator introduced during population initialization accelerates convergence, while the newly proposed Cauchy mutation operator prevents entrapment in local optima and enhances global search capability. Additionally, incorporating an elite evolution strategy further strengthens the algorithm’s global search performance, enabling superior multi-machine collaborative task allocation with balanced task distribution among agricultural machines. CCEVSOA was compared with GA, CSA, WOA, IBES, and SO to validate its performance. Simulation results indicate that task allocation optimized by CCEVSOA achieves substantially lower collaborative operation times and overall fitness values than other algorithms. Implementing CCEVSOA for multi-machine cooperative operations within the same field not only effectively reduces operation time, improves efficiency, and lowers operational costs but also ensures balanced task distribution, thereby laying a solid foundation for the advancement of smart agriculture. Although the simulation results demonstrate CCEVSOA’s significant improvements in multi-machine cooperative task allocation efficiency and its robust optimization performance, limitations related to the current research scope and specific regional conditions indicate opportunities for further refinement. Future work will focus on integrating CCEVSOA into practical agricultural management systems, leveraging innovative agriculture platforms and communication technologies. This includes incorporating real-time sensor data, interfacing with decision-support software, designing communication protocols, and ensuring compatibility with existing farm management systems. Such developments will transition the algorithm from theoretical validation to practical application, enhancing its utility and real-world value.

## References

[1] Wang T, Xu X, Wang C, Li Z, Li D. From smart farming towards unmanned farms: A new mode of agricultural production. Agriculture. 2021;11(2):145. doi: 10.3390/agriculture11020145

[2] Boursianis AD, Papadopoulou MS, Diamantoulakis P, Liopa-Tsakalidi A, Barouchas P, Salahas G, et al. Internet of things (IoT) and agricultural unmanned aerial vehicles (UAVs) in smart farming: A comprehensive review. J IOT. 2020:100187. https://doi.org/10.1016

[3] Alahe MA, Wei L, Chang Y, Gummi SR, Kemeshi J, Yang X. Cyber security in smart agriculture: Threat types, current status, and future trends. Comput Electron Agric. 2024;226:109401.

[4] Zhu A, Zeng Z, Guo S, Lu H, Ma M, Zhou Z. Game-theoretic robotic offloading via multi-agent learning for agricultural applications in heterogeneous networks. Comput Electron Agric. 2023;211:108017.

[5] Cho S, Kim T, Jung DH, Park SH, Na Y, Ihn YS, et al. Plant growth information measurement based on object detection and image fusion using a smart farm robot. Comput Electron Agric. 2023;207:107703.

[6] Lu Z, Wang Y, Dai F, Ma Y, Long L, Zhao Z, et al. A reinforcement learning-based optimization method for task allocation of agricultural multi-robots clusters. Comput Electr Eng. 2024;120:109752.

[7] Li Z, Shi N, Zhao L, Zhang M. Deep reinforcement learning path planning and task allocation for multi-robot collaboration. Alex Eng J. 2024;109:408–23.

[8] Chen T-Y, Miao Z-H, Li W-M, Pan Q-K. A learning-based memetic algorithm for a cooperative task allocation problem of multiple unmanned aerial vehicles in smart agriculture. Swarm Evolut Comput. 2024;91:101694. doi: 10.1016/j.swevo.2024.101694

[9] Bektur G. A reinforcement learning-based multiobjective heuristic algorithm for multiple-truck routing problems with heterogeneous drones. Appl Soft Comput. 2024;167:112290. doi: 10.1016/j.asoc.2024.112290

[10] Yan F, Chu J, Hu J, Zhu X. Cooperative task allocation with simultaneous arrival and resource constraint for multi-UAV using a genetic algorithm. Expert Systems with Applications. 2024;245:123023. doi: 10.1016/j.eswa.2023.123023

[11] Mangalampalli S, Karri GR, Kose U. Multi objective trust aware task scheduling algorithm in cloud computing using whale optimization. J King Saud Univ – Comput Inform Sci. 2023;35(2):791–809. doi: 10.1016/j.jksuci.2023.01.016

[12] Li M, Liu C, Li K, Liao X, Li K. Multi-task allocation with an optimized quantum particle swarm method. Appl Soft Comput. 2020;96:106603. doi: 10.1016/j.asoc.2020.106603

[13] Zhang SH, Wang JS, Zhang SW, Li YX, Xing YX, Zhang YH. Snake optimizer with oscillating factors to solve edge computing task unloading and scheduling optimization problem. Alex Eng J. 2024;91:273–304.

[14] Wang N, Yang X, Wang T, Xiao J, Zhang M, Wang H, et al. Collaborative path planning and task allocation for multiple agricultural machines. Comput Electron Agric. 2023;213:108218.

[15] Gao X, Wang L, Yu X, Su X, Ding Y, Lu C, et al. Conditional probability based multi-objective cooperative task assignment for heterogeneous UAVs. Eng Appl Artif Intell. 2023;123:106404.

[16] Mao W, Liu Z, Liu H, Yang F, Wang M. Research progress on synergistic technologies of agricultural multi-robots. Appl Sci. 2021;11(4):1448.

[17] Li X, Gong Z, Zheng J, Liu Y, Cao H. A survey of data collaborative sensing methods for smart agriculture. Internet of Things. 2024;28:101354. doi: 10.1016/j.iot.2024.101354

[18] Gu Y, Wang Y, Wang T. An approximate dynamic programming approach to dynamic slot allocation of spot containers with random arrivals, cancellations, and no-shows. Transport Res Part E: Logistics Transport Rev. 2025;193:103837. doi: 10.1016/j.tre.2024.103837

[19] Zhen Z, Wen L, Wang B, Hu Z, Zhang D. Improved contract network protocol algorithm based cooperative target allocation of heterogeneous UAV swarm. Aerosp Sci Technol. 2021;119:107054.

[20] Zhao M, Li D. Collaborative task allocation of heterogeneous multi-unmanned platform based on a hybrid improved contract net algorithm. IEEE Access. 2021;9:78936–46. doi: 10.1109/access.2021.3084238

[21] Wang G, Wang F, Wang J, Li M, Gai L, Xu D. Collaborative target assignment problem for large-scale UAV swarm based on two-stage greedy auction algorithm. Aerosp Sci Technol. 2024;149:109146.

[22] Ye X, Guo H, Luo Z. Two-stage task allocation for multiple construction robots using an improved genetic algorithm. Autom Constr. 2024;165:105583.

[23] Xiang J, Zhang Y, Cao X, Zhou Z. An improved multi-objective hybrid genetic-simulated annealing algorithm for AGV scheduling under composite operation mode. CMC-Comput Mater Contin. 2023;77(3):3443–66.

[24] Myriam H, A. Abdelhamid A, El-Kenawy E-SM, Ibrahim A, Eid MM, Jamjoom MM, et al. Advanced meta-heuristic algorithm based on particle swarm and Al-Biruni earth radius optimization methods for oral cancer detection. IEEE Access. 2023;11:23681–700. doi: 10.1109/access.2023.3253430

[25] El-Kenawy E-SM, Khodadadi N, Mirjalili S, Makarovskikh T, Abotaleb M, Karim FK, et al. metaheuristic optimization for improving weed detection in wheat images captured by drones. Mathematics. 2022;10(23):4421. doi: 10.3390/math10234421

[26] Atteia G, M. El-kenawy E-S, Abdel Samee N, M. Jamjoom M, Ibrahim A, A. Abdelhamid A, et al. Adaptive dynamic dipper throated optimization for feature selection in medical data. Comput Mater Continua. 2023;75(1):1883–900. doi: 10.32604/cmc.2023.031723

[27] Abdelhamid AA, El-Kenawy E-SM, Ibrahim A, Eid MM, Khafaga DS, Alhussan AA, et al. Innovative feature selection method based on hybrid sine cosine and dipper throated optimization algorithms. IEEE Access. 2023;11:79750–76. doi: 10.1109/access.2023.3298955

[28] Alkanhel R, El-Kenawy ESM, Abdelhamid AA, et al. Network intrusion detection based on feature selection and hybrid metaheuristic optimization. Comput Mater Continua. 2023;74(2).

[29] Yan F, Di K. Solving the Multi-robot task allocation with functional tasks based on a hyper-heuristic algorithm. Appl Soft Comput. 2023;146:110628. doi: 10.1016/j.asoc.2023.110628

[30] Cao R, Li S, Ji Y, Zhang Z, Xu H, Zhang M, et al. Task assignment of multiple agricultural machinery cooperation based on improved ant colony algorithm. Comput Electr Agric. 2021;182:105993. doi: 10.1016/j.compag.2021.105993

[31] Wang Z, Zhang J. A task allocation algorithm for a swarm of unmanned aerial vehicles based on bionic wolf pack method. Knowl-Based Syst. 2022;250:109072. doi: 10.1016/j.knosys.2022.109072

[32] Guo H, Miao Z, Ji J, Pan Q. An effective collaboration evolutionary algorithm for multi-robot task allocation and scheduling in a smart farm. Knowl-Based Syst. 2024;289:111474. doi: 10.1016/j.knosys.2024.111474

[33] Hashim FA, Hussien AG. Snake Optimizer: A novel meta-heuristic optimization algorithm. Knowl-Based Syst. 2022;242:108320. doi: 10.1016/j.knosys.2022.108320

[34] Haktanirlar Ulutas B, Kulturel-Konak S. A review of clonal selection algorithm and its applications. Artif Intell Rev. 2011;36(2):117–38. doi: 10.1007/s10462-011-9206-1

[35] Mirjalili S, Lewis A. The whale optimization algorithm. Adv Eng Softw. 2016;95:51–67. doi: 10.1016/j.advengsoft.2016.01.008

[36] Abdollahzadeh B, Gharehchopogh FS, Mirjalili S. African vultures optimization algorithm: A new nature-inspired metaheuristic algorithm for global optimization problems. Comput Ind Eng. 2021;158:107408. doi: 10.1016/j.cie.2021.107408

[37] Yu Z, Zhang X, Shen X. An improved differential evolutionary algorithm based on simulated annealing and levy flights mechanism. Eng Lett. 2021;29(2).

